# *Neisseria meningitidis* has acquired sequences within the capsule locus by horizontal genetic transfer

**DOI:** 10.12688/wellcomeopenres.15333.2

**Published:** 2019-08-02

**Authors:** Marianne E. A. Clemence, Odile B. Harrison, Martin C. J. Maiden

**Affiliations:** 1 Department of Zoology, University of Oxford, Oxford, OX1 3SY, UK

**Keywords:** Neisseria, meningitis, capsule, recombination, horizontal genetic transfer, subflava

## Abstract

**Background: **Expression of a capsule from one of serogroups A, B, C, W, X or Y is usually required for *Neisseria meningitidis *( *Nme*) to cause invasive meningococcal disease. The capsule is encoded by the capsule locus, *cps*, which is proposed to have been acquired by a formerly capsule null organism by horizontal genetic transfer (HGT) from another species. Following identification of putative capsule genes in non-pathogenic *Neisseria *species, this hypothesis is re-examined.

**Methods: **Whole genome sequence data from *Neisseria *species, including *Nme *genomes from a diverse range of clonal complexes and capsule genogroups, and non- *Neisseria *species, were obtained from PubMLST and GenBank. Sequence alignments of genes from the meningococcal *cps*, and predicted orthologues in other species, were analysed using Neighbor-nets, BOOTSCANing and maximum likelihood phylogenies.

**Results: **The meningococcal *cps *was highly mosaic within regions B, C and D. A subset of sequences within regions B and C were phylogenetically nested within homologous sequences belonging to *N. subflava*, consistent with HGT event in which *N. subflava *was the donor. In the *cps *of 23/39 isolates, the two copies of region D were highly divergent, with *rfbABC’* sequences being more closely related to predicted orthologues in the proposed species *N. weixii *(GenBank accession number CP023429.1) than the same genes in *Nme *isolates lacking a capsule. There was also evidence of mosaicism in the *rfbABC’* sequences of the remaining 16 isolates, as well as *rfbABC* from many isolates.

**Conclusions: **Data are consistent with the *en bloc* acquisition of *cps* in meningococci from *N. subflava*, followed by further recombination events with other *Neisseria *species. Nevertheless, the data cannot refute an alternative model, in which native meningococcal capsule existed prior to undergoing HGT with *N. subflava *and other species. Within-genus recombination events may have given rise to the diversity of meningococcal capsule serogroups.

## Introduction

*Neisseria meningitidis* ( *Nme*) is a gram-negative bacterium that typically establishes an asymptomatic colonisation of the human nasopharynx. Occasionally, *Nme* invades the bloodstream where, dependent on the possession of certain genetic factors and host-pathogen interactions, it is able to evade immune responses, causing invasive meningococcal disease (IMD) ^1^. IMD usually presents as meningitis and/or septicaemia, which have high mortality rates and are a public health priority in many jurisdictions. Certain clonal complexes (cc) of *Nme*, as determined by seven locus multi-locus sequence typing, represent genetic lineages commonly associated with IMD ^2^. Several genetic factors have been implicated in facilitating the disease phenotype. One factor that is necessary except in very rare cases ^3– 5^ is expression of a polysaccharide capsule belonging to one of serogroups A, B, C, W, X or Y ^6, 7^. A further six serogroups are not associated with disease ^8^.

**Figure 1.**
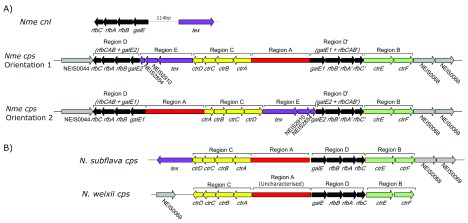
Organisation of *cps*. Organisation of genes within **A**) the two orientations of the meningococcal *cps* and **B**) the *N. subflava* and *N. weixii cps*.

Expression of the meningococcal capsule is ABC transporter-dependent, and the genes required for capsule synthesis (region A) and export of the capsule (regions B and C) are consistently co-located in the chromosome in the capsule locus ( *cps*) ^8^. The capsule genogroup can be determined from region A sequences, enabling inference of the serogroup if capsule is expressed. Also co-located in *cps* is region D, which consists of *galE* and *rfbABC*, and region D’, a duplicated version of region D. The gene *galE* has been shown to be involved in LPS synthesis ^9^, and is also necessary for the synthesis of the capsule in serogroups E and Z ^10, 11^. There is dynamic inversion of genes within the capsule locus between *galE1*, and the truncated gene *galE2*, giving rise to two capsule orientations ( Figure 1A) ^12^. It has been noted that, since the *cps* is located 54 kb downstream of the origin of replication, it is possible that these inversions resolve collisions between transcription and genome replication machinery ^12^, as described in *Escherichia coli* ^13^, which may be important in regions where genes are highly expressed. Region E consists of the putative transcriptional accessory protein *tex,* a modification methyltransferase, and a truncated adenine-specific methyltransferase, none of which have been implicated in capsule synthesis ^8^. Flanking the 3’ end of region B is an additional hypothetical gene designated as NEIS0068 ^12^.

There are several meningococcal ccs, including cc198, cc53, cc192, cc1117 and cc1136, that are consistently found to lack genes required for capsule synthesis. Isolates lacking a capsule are designated capsule null ( *cnl*) ^14^. Isolates of the *Neisseria* species most closely related to *Nme*, including *N. gonorrhoeae, N. polysaccharea* and *N. lactamica* are also consistently *cnl* ^15^. None of regions A, B, or C are found in these capsule null isolates, and they only possess one copy of region D.

The absence of a *cps* in the closest relatives of *Nme* led to the proposal that meningococcal *cps* may have been acquired as a result of a horizontal genetic transfer (HGT) event, resulting in the duplication of region D ^12, 15, 16^. A member of the *Pasteurellacae* family was proposed as a possible donor, based on sequence identity between capsule export genes in *Pasteurella multocida* and *Nme* ^17^. The recent discovery of capsule genes in non-pathogenic *Neisseria* species, including the human-associated species *N. subflava, N. elongata* and *N. oralis,* supported the more likely explanation that the capsule was lost from an ancestor of *Nme*, and then later reacquired from another *Neisseria* species ^15^. *Nme* is highly competent for transformation by HGT, and co-exists in the nasopharynx with many other *Neisseria* species, as well as bacteria from other genera. In addition to frequent recombination events within meningococcal populations, there have been several accounts of HGT from non-pathogenic *Neisseria* species to *Nme*, including genes associated with virulence and antibiotic resistance ^18– 23^.

The analysis reported here reveals a complex evolutionary history of meningococcal *cps,* involving multiple HGT events with other *Neisseria* species, one of which was likely *N. subflava.* The data reported are consistent with models hypothesising that the meningococcal capsule locus was acquired *en bloc* ^12^ by HGT into a capsule null organism.

## Methods

### Isolate collection

Meningococcal whole genome sequencing data (WGS) with good *cps* assembly were chosen from the Meningitis Research Foundation Meningococcus genome library (consisting of UK disease-associated isolates), the meningococcal 107 global collection project (consisting mostly of disease-associated isolates) ^24^, and a UK carriage dataset collected by the University of Oxford, all of which are available from https://pubmlst.org/neisseria/, hosted on the Bacterial Isolate Genome Sequence Database (BIGSdb) genomics platform ^25^. Meningococcal genomes were chosen at random from the datasets to provide up to one cc/genogroup combination from both carriage and disease where available. WGS from additional public pubMLST isolates M01-240355 ^26^ and WUE2594 ^27^ were chosen to include cc213 and cc5, respectively. Additional *cps* sequence data from isolates with capsule genogroup E, L, W, X or Z were retrieved from GenBank ^28^, originating from characterisation of meningococcal capsule serogroups ^8^.

WGS data from representative isolates of other *Neisseria* species were sourced from pubMLST, including the novel species *Neisseria weixii* (strain 10022, GenBank accession number CP023429.1). WGS from non- *Neisseria* species were sourced from GenBank and chosen for the presence of genes homologous with those within the meningococcal *cps*. including *Actinobacillus succinogenes* (strain 130Z, GenBank accession number CP000746.1), *Actinobacillus pleuropneumoniae* (AP76, CP001091.1), *Aggregibacter actinomycetemcomitans* (D11S-1, CP001733.2), *Bibersteinia trehalosi* (USDA-ARS-USMARC-189, CP006955.1), *Vibrio vulnificus* (NBRC 15645, CP012881.1), *Glaesserella* sp. (15-184, CP023057.1), *Actinobacillus porcitonsillarum* (9953L55, CP029206.1), *Haemophilus influenzae* (18010, FQ312006.1), *Kingella kingae* (KW1, LN869922.1) and *Actinobacillus suis* (NCTC12996, LT906456.1). The full isolate collection dataset is available as *Extended data* ^29^.

Speciation was confirmed using ribosomal multi-locus sequence typing (rMLST) ^30^. Loci defined within the rMLST scheme, with the exception of *rpmE* and *rpmJ*, which are duplicated in some *Neisseria*, were extracted and aligned with MAFFT ^31^ within the BIGSdb genome comparator module. TrimAl ^32^ was used to remove sites with gaps in more than 10% of sequences. A neighbor-joining tree was generated with the Jukes-Cantor ^33^ substitution model using phangorn (v2.4.0) ^34^ implemented in R, and rooted at the mid-point.

### Annotation of capsule loci

The majority of the *Neisseria* WGS in pubMLST had previously been fully annotated manually for *cps* genes *rfbABC* (NEIS0045-7), *galE* (NEIS0048), *tex* (NEIS0059), the pseudo-methyltransferases NEIS2854 and NEIS2910 *, ctrABCDEF* (NEIS0055-8), and flanking genes NEIS0044, NEIS0068 and NEIS0069, where present. Genomes in which one or more of these genes had not been annotated were queried using the BLASTn-based scanning tool in pubMLST; if a relevant gene was identified, this was tagged in the WGS data and an appropriate allele designation set. *Nme* sequences from GenBank had a fully annotated *cps* ^8^. Predicted orthologues of *cps* genes were identified in non- *Neisseria* species using BLASTn.

The meningococcal genomes possessing a capsule were investigated to determine whether the capsule locus was in orientation 1 (-NEIS0044-><- *rfbCAB*-<- *galE2*-<-Region E-<-Region C--Region A-- *galE1*->- *rfbBAC’*->-Region B->-NEIS0068->-NEIS0069->) or orientation 2 (-NEIS0044-><- *rfbCAB*-<- *galE1*—Region A—Region C->-Region E->- *galE2*->- *rfbBAC’*->-Region B->-NEIS0068->-NEIS0069->) ( Figure 1A). *galE1/2* were distinguished according to the nomenclature used by Bartley *et al.* ^12^, in which the truncated form of *galE* within *cps* was designated *galE2*, and the full length form within *cps* as *galE1*. If the capsule locus spanned more than one assembled contig, the orientation was assumed based on the co-localisation of the relevant genes and regions.

### Sequence alignment and phylogenetic analysis

Gene sequences were exported from pubMLST from meningococcal and non-meningococcal *Neisseria* WGS. Sequences were downloaded manually from GenBank from non- *Neisseria*. Amino acid sequences were deduced in MEGA X ^35^ and aligned using Muscle ^36^, correcting for frameshift mutations where applicable, and manually trimmed to give a final nucleotide sequence alignment.

Aligned nucleotide sequences of region C genes *ctrABCD*, region B genes *ctrEF,* and *rfbABC*, or predicted homologues, were concatenated separately. Full length *galE/galE1* orthologues were also analysed separately. Each concatenated set of sequences was loaded into SplitsTree4 (v4.14.9) ^37^ and a phylogenetic network was deduced using the neighbor-net algorithm ^38^. Groups were identified based on a balance between maximising edge weighting, whilst minimising contradictory splits.

Sequences from meningococcal isolates CA41967, Z2491, α707, WUE171 and 1.02397.V were chosen for further investigation of the whole capsule locus and its flanking regions, and compared to sequences from ST42119 (capsule null *Nme*), NJ9703 ( *N. subflava*) and 10022 ( *N. weixii*), with USDA-ARS-USMARC-188 ( *B. trehalosi*) included as an outgroup. Aligned nucleotide sequences of *rfbCAB+galE2, ctrDCBA, rfbBAC’+galE1, ctrEF,* and NEIS0069 (where sequenced), were concatenated separately, since capsule null *Nme* does not contain *ctrDCBA* or *ctrEF*; gene sequences were orientated to be in the same direction as they would be relative to NEIS0044 in orientation 1 of the meningococcal *cps* ( Figure 1A), where NEIS0044 is in the forward orientation. Each concatenated set of sequences was loaded into the Recombination Detection Programme 4 ^39^. Recombination was assessed in each *cps ^+^* meningococcal isolate using manual BOOTSCANing ^40^, with capsule null *Nme*, *N. subflava, N. weixii* and *B. trehalosi* as reference sequences. Neighbor-joining trees were used with the Jukes-Cantor substitution model and 100 bootstraps. Bootstrap support below 70% was disregarded. In order to minimise false breakpoints that may occur due to high sequence identity, appropriate window size was determined by testing CA40160, CA41628 and GL40098 (capsule null *Nme*), and OX42005 ( *N. subflava*), which were not expected to have recombinant capsule sequences. Window size was set at 400 bp for *rfbABC*+ *galE1/2,* 200 bp for *ctrDCBA* and *ctrEF*, and 250 bp for NEIS0069. Step size was set at 10% of window size.

Aligned nucleotide sequences of *ctrEF*, less the first 774 bp of *ctrE*, which were suspected to be recombinant, were concatenated. A maximum likelihood phylogeny of *Neisseria* sequences, with *B. trehalosi* as an outgroup, was generated in PhyML (v3.1) ^41^ with 100 bootstraps, using the GTR+I+G substitution method ^42^, determined to be the best fit by jModelTest (v2.1.10) ^43^. A second phylogeny was generated in the same way using aligned *ctrD* nucleotide sequences that were suspected to be non-recombinant.

## Results

### Species confirmation and annotations

**Figure 2.**
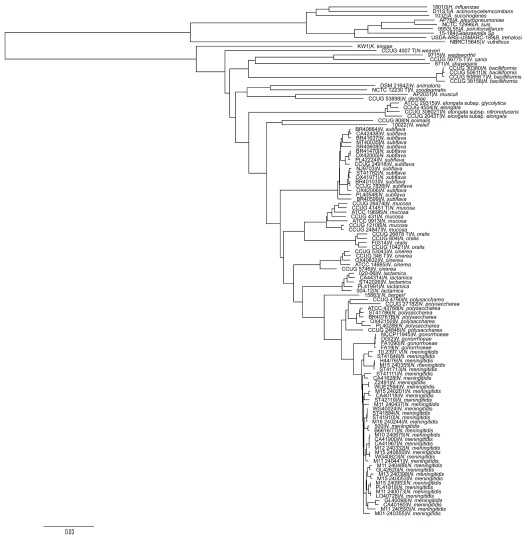
Species phylogeny. Neighbor-joining tree using the Jukes-Cantor substitution model, generated from concatenated, aligned rMLST nucleotide sequences.

Designated species names matched their position in a phylogeny based on rMLST, with sequences from all *Neisseria* isolates belonging to a single clade ( Figure 2). The distribution of capsule export genes *ctrABCDEF*, region D genes *rfbABC* + *galE* and NEIS0059 was consistent with previous descriptions ^8, 15^, and all 11 genes were also annotated in the proposed species *N. weixii*, which was also observed to contain homologues of the putative region A from the *N. animalis cps* ( Figure 1B). Additionally, the 345 bp pseudo-methyltransferase NEIS2854 was present within all *cps* ^+^ meningococci, as well as the capsule null strain ST42119 (cc198); NEIS2854 is a truncated version of the 1008 bp gene NEIS2725, which was only present in WGS from *N. gonorrhoeae* isolates and one *N. polysaccharea* isolate (CCUG 4790) only. The pseudo-methyltransferase NEIS2910 was present in all *cps* ^+^ *Nme*, ST42119, *N. gonorrhoeae* and CCUG 4790 genomes. The hypothetical gene NEIS0068, which flanks region B of the capsule locus, was identified within genomes from all *cps* ^+^ *Nme* and *N. subflava* isolates, but no other *Neisseria* species or capsule null meningococci. The organisation of the N. subflava cps was described previously ^15^ ( Figure 1B).

### Regions B and C of the meningococcal *cps* are mosaic

Phylogenetic analyses of meningococcal *cps* regions B and C, along with predicted orthologues from other *Neisseria* species and proteobacteria, were consistent with the presence of recombinant sequences in the meningococcal *cps*.

**Figure 3.**
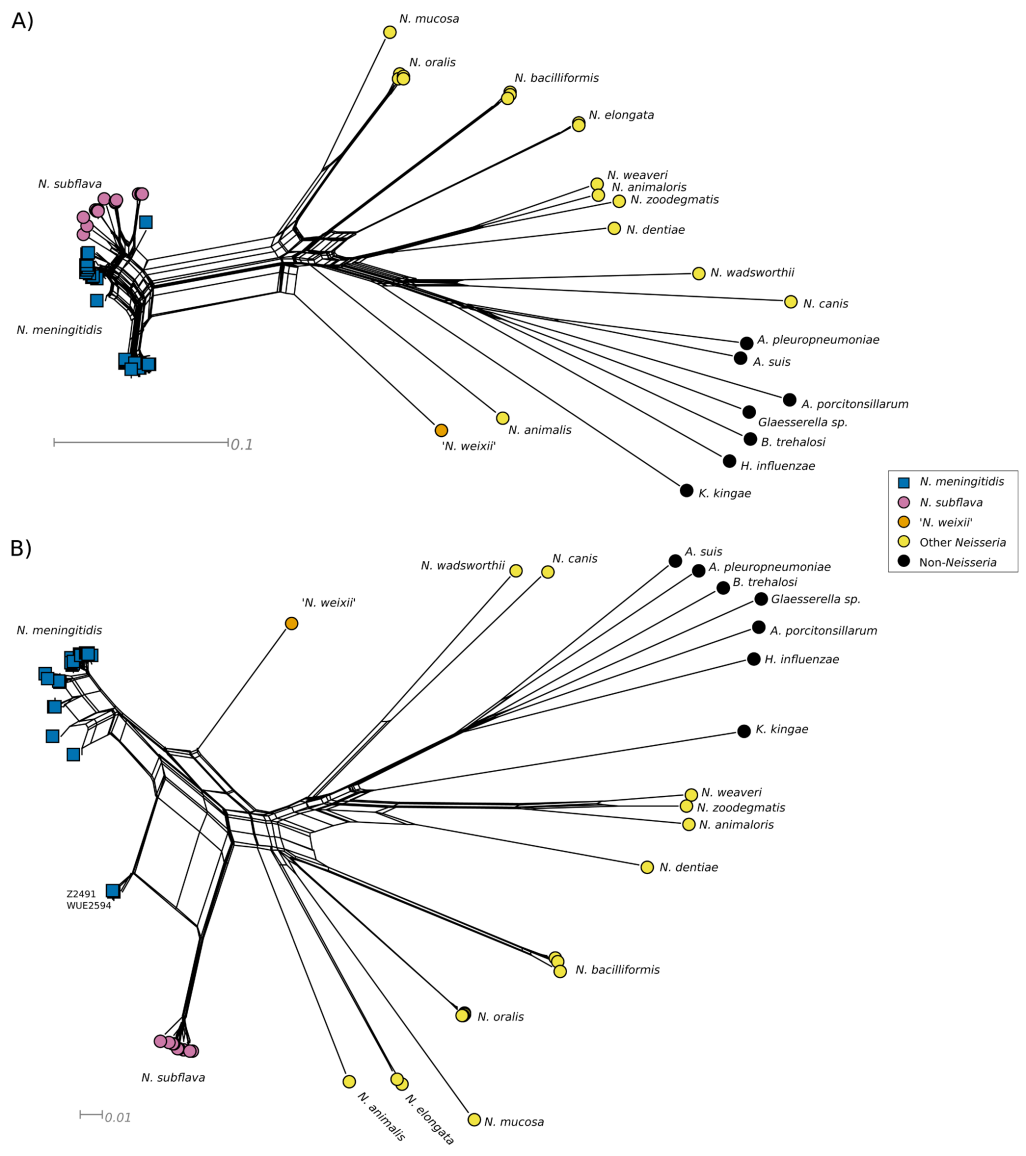
Region B and C neighbor-nets. Neighbor nets generated using concatenated, aligned nucleotide sequences of ( **A**) region B and ( **B**) region C genes. Edges represent splits that support the separation of two clusters in the network, with the length of the line representing the weight of the split. Increasing number of parallel edges represents contradictory splits.

**Figure 4.**
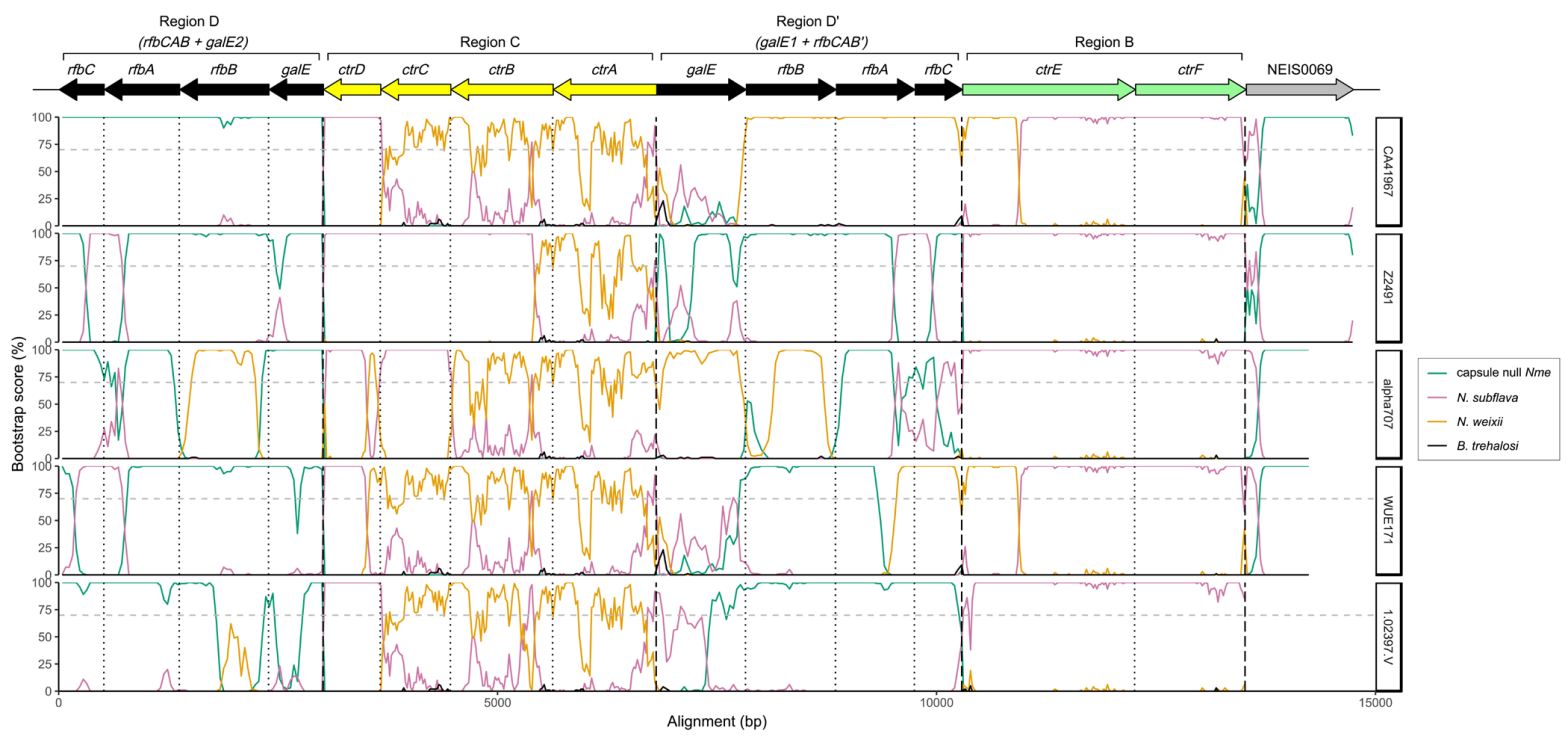
BOOTSCANs of *cps*. Recombination analysis of chosen *cps* sequences from CA41967, Z2491, α707, WUE171 and 1.02397.V by BOOTSCANing, with ST42119 (capsule null *Nme*), NJ9703 ( *N. subflava*), 10022 ( *N. weixii*) included as potential parent sequences, and USDA-ARS-USMARC-189 ( *B. trehalosi*) included as an outgroup. Vertical short dashed lines represent gene boundaries, and vertical long dashed lines represent separate analyses.

Neighbor-net analysis revealed a well-supported split that grouped region B sequences from meningococci and *N. subflava* together ( Figure 3A). There was also some support for a contradictory split grouping region B sequences from 24 of the meningococci with *N. weixii*. BOOTSCANing in 200 bp windows of CA41967, one of the 24, was consistent with a mosaic Region B in the *cps*, with at least the first 774 bp of *ctrE* having support for *N. weixii* as the nearest neighbour grouping, before switching to *N. subflava* ( Figure 4).

A neighbor-net generated from region C sequences contained several contradictory splits that either grouped meningococcal region C sequences with *N. subflava* or the proposed species *N. weixii*, both with high support relative to the rest of the network. There was no well-supported split clustering all three into a single group ( Figure 3B). This was corroborated by BOOTSCANing of representative isolates: all five *cps* sequences scanned had good bootstrap support for *N. subflava* as the nearest neighbour grouping for at least the first 440 bp of region C (at the 3’ end of *ctrD*) ( Figure 4). Across the rest of the region, there was greater bootstrap support for *N. weixii* as the nearest neighbour, although the signal was noisy. In Z2491, the signal for *N. subflava* extended for 2379 bp (comprising *ctrD, ctrC* and much of *ctrB*) before switching to *N. weixii.* This was consistent with a relatively highly weighted neighbor-net split which grouped Z2491, as well as WUE2594, with *N. subflava*.

### Meningococcal capsule export sequences are nested within homologous sequences in *N. subflava*

**Figure 5.**
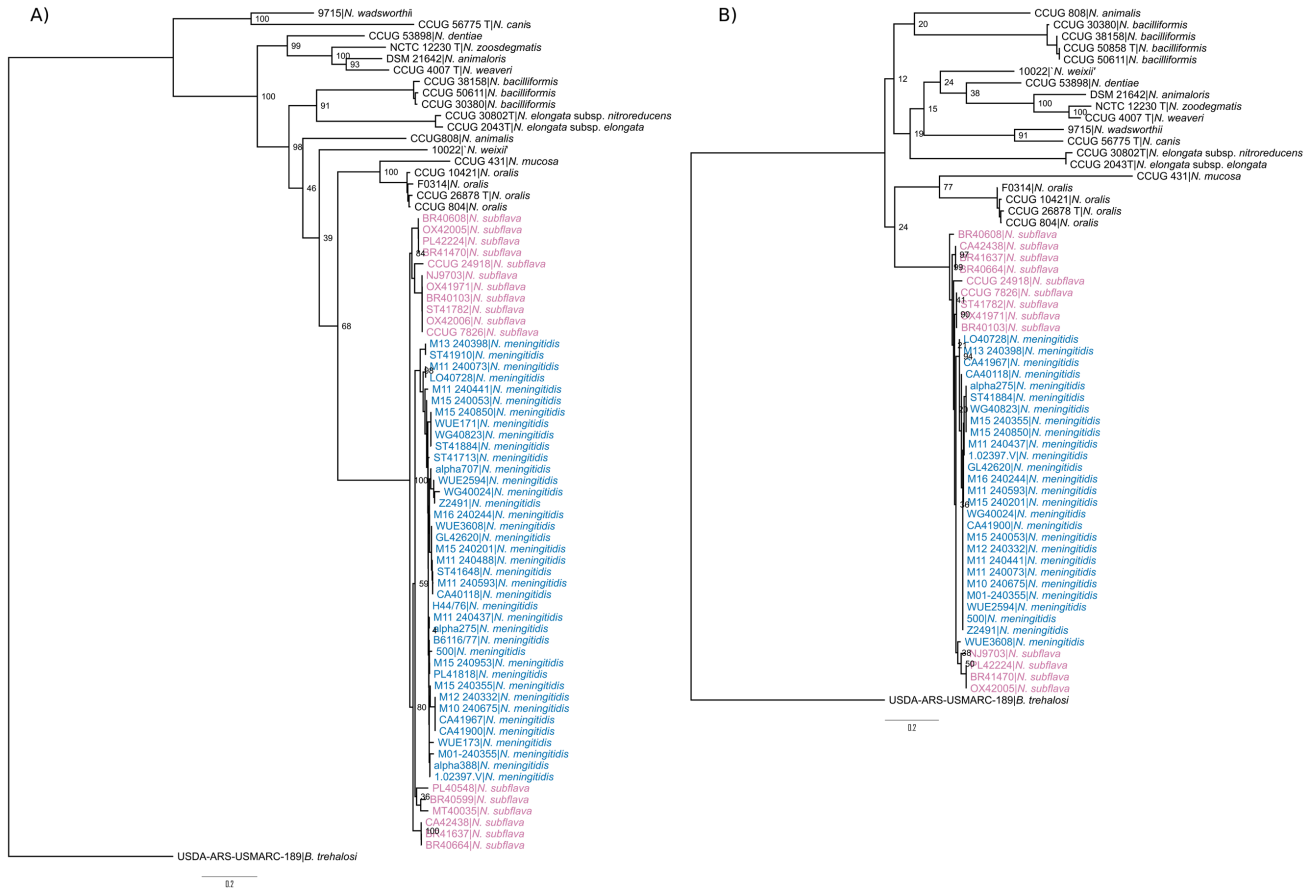
Region B and *ctrD* phylogenies. Maximum likelihood phylogenies using the GTR+I+G substitution model, generated from concatenated, aligned nucleotide sequences of ( **A**) region B genes *ctrEF* (NEIS0066 was trimmed at the 5’ end to remove potentially recombinant sequences) and ( **B**) *ctrD*. In both phylogenies, *B. trehalosi* was included as an outgroup; *Nme* blue, *N. subflava* pink.

A maximum likelihood phylogeny of region B, excluding the first 774 bp of *ctrE*, which was determined by BOOTSCANing to be potentially recombinant in some WGS, revealed that region B sequences from meningococci are nested within homologous sequences from *N. subflava* ( Figure 5A). The diversity of these sequences was much lower in *Nme* (mean p-distance 0.017) than *N. subflava* (mean p-distance 0.040). Similarly, suspected non-recombinant *ctrD* sequences from meningococci were nested within predicted homologous sequences belonging to *N. subflava* ( Figure 5B). 

### Region D’ of the *cps* locus is not a duplication of meningococcal region D

As described previously ^12^, the meningococcal genomes possessed the *cps* locus in either the (-NEIS0044-><- *rfbCAB*-<- *galE2*-<-Region E-<-Region C--Region A-- *galE1*->- *rfbBAC’*->-Region B->-NEIS0068->-NEIS0069->) orientation, or the (-NEIS0044-><- *rfbBAC*-<- *galE1*—Region A—Region C->-Region E->- *galE2*->- *rfbBAC’*->-Region B->-NEIS0068->-NEIS0069->) orientation ( Figure 1A). *galE1* was distinguished from *galE2* by the fact that the latter is consistently truncated at the 5’ end.

**Figure 6.**
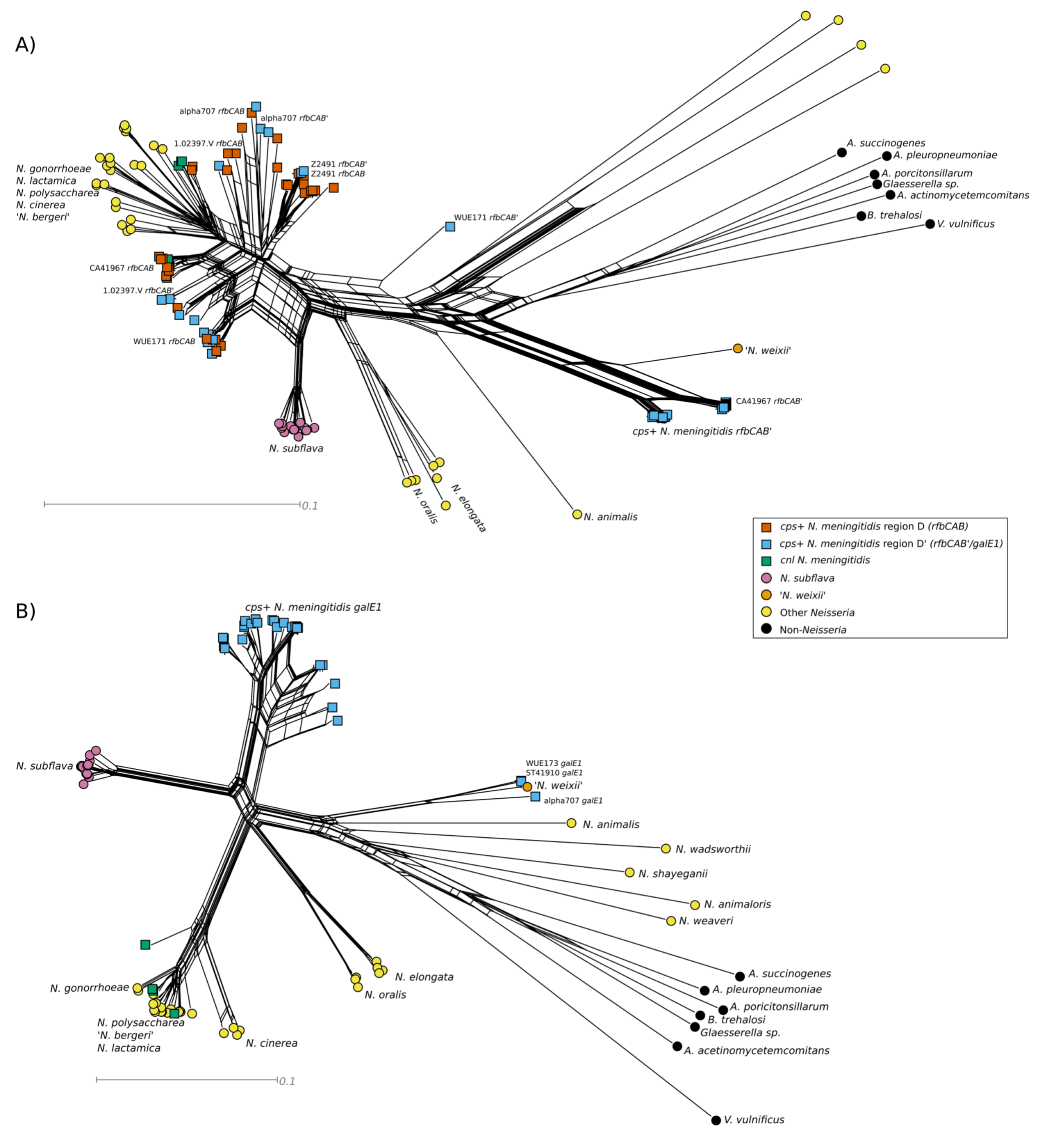
Region D neighbor-nets. Neighbor nets generated using concatenated, aligned nucleotide sequences of ( **A**) *rfbABC* genes, with both *rfbABC* and *rfbABC’* extracted from *cps+* meningococci, and ( **B**) full length *galE* or *galE1* (truncated *galE2* alleles not included).

Phylogenetic analyses of *rfbABC’*, along with predicted orthologues from other *Neisseria* species and proteobacteria, were consistent with acquisition of *rfbABC’* sequences by HGT from another *Neisseria* species ( Figure 6A). In WGS from 23 isolates, neighbor-net splits supported the grouping of *rfbABC’* with homologous sequences from the novel species *N. weixii*. This relationship was further supported by BOOTSCANing of concatenated *rfbBAC’* sequences from CA41967, which showed high bootstrap support for *N. weixii* as the nearest neighbour grouping across most of the region ( Figure 4). There was a drop in bootstrap support for any reference sequence across *galE1*, possibly indicating the absence of a good representative reference sequence. An appropriate sequenced reference could not be identified using a neighbour-net generated from aligned *galE, galE1* and predicted orthologues ( Figure 6B), or by searching the NCBI nucleotide collection. *galE1* sequences from α707 (serogroup E), WUE173 (serogroup Z) and ST41910 (serogroup Z) grouped with predicted orthologous sequences in *N. weixii*, consistent with BOOTSCANing analyses of α707 ( Figure 4).

There were no highly weighted splits supporting the grouping of the sequences from the remaining 16 isolates with any other species, and a high degree of reticulation was present within the neighbor-net, which can be indicative of recombination ( Figure 6A). This was consistent with BOOTSCANing results in sequences from chosen isolates ( Figure 4). Z2491 *rfbBAC*’ + *galE1* contained sequences similar to both capsule null *Nme* and *N. subflava*. α707 and WUE171 *rfbBAC’* contained sequences similar to both *Nme* and *N. weixii*, with a drop off in bootstrap support within WUE171 sequences across *galE1.* The 1.02397.V *rfbBAC’* sequences were similar to capsule null *Nme*, again with a drop-off in bootstrap support for any reference across *galE1*.

Neighbor-net analysis of *rfbABC* sequences contained a split supporting the grouping of the concatenated sequences with capsule null meningococci, but again there was a lot of reticulation ( Figure 6A). Sequences from chosen isolates were investigated further using BOOTSCANing. BOOTSCAN results of *rfbABC* + *galE2* sequences from Z2491 and WUE171 were consistent with a recombination event involving *N. subflava* ( Figure 4). In 1.02397.V, there was only bootstrap support for capsule null *Nme*, although there was a drop-off in bootstrap support through parts of *rfbB* and *galE2*. α707 *rfbABC* + *galE2* sequences were grouped with the capsule null group according to the split, but mosaic signals were identified in the sequences consistent with a recombination event involving a species more closely related to *N. weixii.* CA41967 specifically clustered with capsule null *rfbABC* sequences, consistent with BOOTSCANs which did not demonstrate recombination in this region.

## Discussion

In this analysis, evidence is presented that is consistent with a donation of capsule export gene sequences from *N. subflava* to the meningococcal *cps,* as has been previously postulated ^15^. Following the identification of recombinant sequence data ( Figure 3 and Figure 4), further analyses demonstrated that non-recombinant tracts within regions B and C were phylogenetically nested within homologous *N. subflava* sequences ( Figure 5). This phylogenetic pattern would only be expected to occur if the true donor was a member of *N. subflava*, rather than another species closely related to *N. subflava*. HGT is more likely to occur between closely related species, since higher sequence identity facilitates homologous recombination. *N. subflava* is also widely carried by humans, and it has previously been suggested that strains of the close relative of *Nme, N. gonorrhoeae,* may have acquired *penA* genes associated with antimicrobial resistance from *N. subflava* through HGT ^44^. The tissue tropism of the *Nme* and *N. subflava* are slightly different, with *N. subflava* isolated more commonly from the buccal cavity than the nasopharynx ^45^, but both are frequently isolated from carriage studies using pharyngeal swabs ^46, 47^. Therefore, HGT of *cps* sequences between *N. subflava* and *Nme* is biologically conceivable.

Sequence analyses indicate that a second species donated sequences within region C of all meningococci analysed, and region B of several meningococcal isolates, resulting in mosaic loci ( Figure 3 and Figure 4). If any region B or C sequences were acquired by the meningococcus by descent, they could not be identified in these sequence analyses ( Figure 4). The novel species *N. weixii* ^48^ was identified as a possible candidate ( Figure 3 and Figure 4), described as being isolated from the Tibetan Plateau Pika ( *Ochotona curzoniae*) in the Qinghai Province, China (GenBank accession CP023429.1). This isolate contained sequences homologous to the *N. animalis* putative region A between its region C and region D homologues ( Figure 1B). Epidemiological interaction between *N. weixii* and *Nme* is unlikely, since the pika is a member of the *Lagomorpha* found in alpine meadows. Analyses by rMLST ( Figure 2) indicate that the next closest species is the guinea pig-associated species *N. animalis*; all sequenced human-associated species are relatively distantly related. An as-yet-unidentified human-associated *Neisseria* species more closely related to *N. weixii* could be the donor of these sequences.

In many of the meningococcal genomes analysed, results were also consistent with HGT of duplicated region D sequences. In these genomes, *rfbABC’+ galE1* sequences were divergent from *rfbABC+ galE* sequences belonging to capsule null meningococci, either in whole or in part ( Figure 6A). BOOTSCANing analyses were consistent with either *N. subflava* or something related to *N. weixii* being the candidate donor of the divergent *rfbBAC’* sequences ( Figure 4). The *galE1* sequences of α707 (cc254, genogroup E), ST41910 (cc1157, genogroup Z) and WUE173 (genogroup Z) also clustered with *N. weixii* ( Figure 6B). In all other meningococcal genomes, *galE1* was divergent from *N. subflava, N. weixii* and capsule null *Nme*, and the donor of this sequence may be an as yet unidentified *Neisseria* species ( Figure 6B). The divergence between *galE1, galE2* and capsule null *galE* sequences, including genogroup E and Z outliers, has been described previously by Bartley *et al.* ^12^. In the same study, it was also demonstrated that *galE* is bi-functional, synthesising UDP-galactose and UDP-galactosamine, and *galE2* is a truncated gene closely related to *galE. galE1* was determined to be predominantly mono-functional, producing only UDP-galactose, but bi-functional in genogroups Z and E, which require this bi-functionality for capsule synthesis. The current study has so far discussed evidence indicating that the meningococcal *cps* is highly mosaic, having undergone HGT with as many as three other *Neisseria* species, but Bartley *et al.* go further, proposing that the phylogenetic distribution and functionality of *galE1* and *galE2* could be explained by the process of an *en bloc* transfer of the entire capsule locus from a donor species into modern meningococcal clones ^12^.

The hypothesis that the meningococcal *cps* was acquired *de novo* by a previously capsule null meningococcal recipient, as a result of a HGT event, has been proposed several times ^12, 15– 17, 49^. Such an event would have had important consequences on the epidemiology of *Nme*, since the possession of a capsule is almost always necessary for IMD ^6, 7^. The existence of the *H. influenzae* capsule has also invoked HGT from another species. Similarly to *Nme*, *H. influenzae* consists of variants both with and without a capsule; the capsule locus *cap* was proposed to have been donated by HGT from *Haemophilus sputorum*, although *H. sputorum* may actually be a member of another genus from the *Pasteurellacae* family ^50^. The low GC content of region A of the capsule locus has also been cited as evidence that the capsule may have been acquired by HGT ^8, 51^, but this has also been observed in the capsule synthesis region of other *Neisseria* species ^15^, as well as *E. coli*, *H. sputorum* and *A. pleuropneumoniae* ^52– 54^. Therefore, GC content may not directly inform on the recent evolutionary history of the meningococcal capsule. The sequence identity between capsule export sequences in *Nme* and *P. multocida* formed the basis of a hypothesis invoking donation of the capsule by a member of the *Pasteurellacae* family, in the absence of further *Neisseria* WGS data at the time ^17^. More recent data demonstrate that capsule export gene sequences are more closely related to homologous sequences from non-pathogenic *Neisseria* species ^15^ ( Figure 3), which raises the question as to whether the capsule was simply inherited by descent. The rationale for a *de novo* acquisition of capsule into a capsule null clone was presented based on the distribution of capsule genes throughout the *Neisseria* genus, with capsule genes not having been identified in any isolates of the species most closely related to *Nme*: *N. gonorrhoeae*, *N. polysaccharea*, *N. lactamica*, *N. cinerea* and ` *N. bergeri’*, which may have resulted from a loss of capsule in a common ancestor of these species, followed by reacquisition in *Nme* ^15^.

The validity of the *en bloc* acquisition model has been further tested using a genome dataset containing a wide diversity of meningococcal clonal complexes and capsule genogroups. BOOTSCANing analyses consistently support *N. subflava* as the nearest neighbour grouping at both ends of the capsule locus, and perhaps into NEIS0069 ( Figure 4). The *en bloc* model also postulates that the donor capsule locus was arranged <-NEIS0059-<-Region C-Region A->-Region D->-NEIS0068->-NEIS0069-> ^12^, which is the same arrangement as characterised in *N. subflava* ^15^, and the flanking gene NEIS0068 has only been found in *cps* ^+^ meningococci and *N. subflava*. The arrangement of orientation 1 of the meningococcal *cps* and the *N. subflava cps* between *tex* and NEIS0069 is equivalent ( Figure 1). These observations are consistent with, but not proof of, an *en bloc* donation of capsule from *N. subflava,* as opposed to a member of another genus, to a capsule null meningococcal clone, with subsequent recombination events with at least two other species.

An alternative explanation is that a duplication of meningococcal region D, and perhaps the whole capsule locus, previously existed in the recipient organism. This alternative explanation could account for the fact that some isolates still contain sequences resembling capsule null meningococci in region D’ ( Figure 4 and Figure 6A). This may be explained by dynamic inversions of the capsule locus ( Figure 1A) ^12^. If inversions involve multiple recombination events with different break points, sequences within the two regions could become unlinked, making it difficult to trace their evolutionary history, and erasing evidence of an acquisition event within region D’ sequences. This problem is exacerbated by capsule switching that occurs between meningococcal clones, wherein HGT events do not necessarily involve the capsule locus in its entirety ^55^. With these issues, by the nature of the question ^56^, and the relatively small size of WGS datasets compared to global *Neisseria* populations through time, it would be difficult to prove beyond doubt that the meningococcal capsule was acquired *de novo* by a capsule null clone, unless a meningococcal isolate were identified with a complete *N. subflava* capsule locus, requiring an absence of further recombination events. Unravelling the complete evolutionary process that led to the modern-day *cps* may not be possible using only contemporary meningococcal genomes.

An outstanding question concerns the origins of region A. Neither *N. weixii* nor *N. subflava* possess putative region A sequences that are highly comparable to those found in meningococcal capsule serogroups, although they do share some homologous sequences. Recombination events that include region A capsule synthesis genes, which result in capsular serogroup switching, have been repeatedly observed within meningococcal populations ^55, 57, 58^. The results presented here raise the question as to whether meningococcal serogroup diversity may have arisen through capsule switching with other species. The serogroup B capsule is structurally equivalent to that of *E. coli* K1, *Mannheimia haemolytica* A2 and *Moraxella nonliquifaciens* ^59^. There is homology between the synthesis regions of these capsules ^60^, but amino acid sequence identity above 70% has not been reported, and sequence data did not show evidence of non- *Neisseria* sequences having been donated to region C or region D ( Figure 3, Figure 4). *galE1* sequence clustering indicate that an un-sequenced Neisseria species may have donated sequences to *Nme* ( Figure 6B). It may be informative to characterise putative region A homologues of any new species identified, which may shed further light on this question.

In conclusion, WGS data are consistent with a model whereby the meningococcal capsule locus was acquired by a capsule null meningococcal clone *en bloc* in a HGT event from a single donor, most likely *N. subflava.* Subsequent homologous recombination events with at least two other species resulted in a highly mosaic locus. Nevertheless, these data are insufficient to rule out an alternative model, in which native meningococcal capsule existed prior to undergoing HGT with *N. subflava* and other species. It is possible that serogroup diversity of meningococcal populations increased as a result of cross-species HGT events. Characterisation of putative capsule genes of newly isolated *Neisseria* species, particularly those isolated from humans, may provide new insights into the complex evolutionary history of the meningococcal capsule locus.

## Data availability

### Underlying data

Details on the sequences of isolates used in the present study, obtained from PubMLST and GenBank, are available as *Extended data*.

### Extended data

Figshare: Isolates used in "Neisseria meningitidis has acquired sequences within the capsule locus by horizontal genetic transfer". https://doi.org/10.6084/m9.figshare.8256572.v1 ^29^.

This project contains the full list of isolates used in this study, along with associated metadata, and pubMLST ID and/or accession number.

Extended data are available under the terms of the Creative Commons Zero "No rights reserved" data waiver (CC0 1.0 Public domain dedication).
