# Supplementary material for: Neisseria meningitidis has acquired sequences within the capsule locus by horizontal genetic transfer
Source: Wellcome Open Res. 2019 Aug 2;4:99. Originally published 2019 Jun 21. [Version 2] doi: 10.12688/wellcomeopenres.15333.2 (PMC6619384; doi:10.12688/wellcomeopenres.15333.2)
Supplement: Peer review history

# Peer review: Reviewer response for version 2

**DOI:** 10.21956/wellcomeopenres.16805.r36108

**Published:** 2019-08-02

**Authors:** H. Steven Seifert

**Affiliations:** 1 Department of Microbiology–Immunology, Northwestern University Feinberg School of Medicine, Chicago, IL, USA

No comments

I confirm that I have read this submission and believe that I have an appropriate level of expertise to confirm that it is of an acceptable scientific standard.

# Peer review: Reviewer response for version 1

**DOI:** 10.21956/wellcomeopenres.16741.r35815

**Published:** 2019-07-08

**Authors:** H. Steven Seifert

**Affiliations:** 1 Department of Microbiology–Immunology, Northwestern University Feinberg School of Medicine, Chicago, IL, USA

This manuscript expands on previous analyses, many by some of the same authors, which examines the evolutionary history of different portions of the capsule locus of *Neisseria meningitidis (Nme). *This analysis uses a greater number of genomic sequences and adds the *Neisseria weixii *species into the analysis. There is also a detailed analysis of possible recombination events in different portions of the locus. They support the previously set forth hypothesis that the capsule locus was acquired by *Nme* by horizontal gene transfer (HGT) from another organism and strongly suggest the donor organism was *Neisseria subflava. *They also put forth an alternative hypotheses that the capsule locus was present in a progenitor organism to *Nme* and has diversified through HGT. Considering previous analyses have suggested that the predecessor of *Nme *and *Neisseria gonorrhoeae *arose from a *N. subflava *lineage is not fully discussed, and appears to support the secondary hypothesis (Donati et al, Nature Microbiol. 2016, Cited Ref 46). As the authors point out, it is impossible to distinguish between these evolutionary models with these types of retrospective data. Possibly the text favors the HGT hypothesis because its previously been proposed many times. It might make more sense at this time to propose that both hypotheses have equal weight. Regardless of this quibble, this work highlights the recent evolutionary history of the genes expressing this essential virulence factor.

I confirm that I have read this submission and believe that I have an appropriate level of expertise to confirm that it is of an acceptable scientific standard.

# Response

**Published:** 2019-07-26

**Authors:** Marianne Clemence

**Affiliations:** University of Oxford, UK

We thank the reviewer for his comments and interest in the article.

 In response to comments in the final sentences: we have used the robust rMLST scheme (1) for speciation that does not reveal a sublineage within *N. subflava *(2). Nevertheless, we have tried to avoid asserting weight to either hypothesis by only going so far as to say that data “are consistent with” an *en bloc *hypothesis. In version 2, we have now emphasised that these data do not provide proof (discussion paragraph 5) of the *en bloc *model.

 1. Jolley KA, Bliss CM, Bennett JS, Bratcher HB, Brehony C, Colles FM, Wimalarathna H, Harrison OB, Sheppard SK, Cody AJ, Maiden MCJ. Ribosomal multilocus sequence typing: universal characterization of bacteria from domain to strain. Microbiology. 2012;158:1005-15

 2. Bennet JS, Jolley KA, Earle SG, Corton C, Bentley SD, Parkhill J, Maiden MCJ.  A genomic approach to bacterial taxonomy: an examination and proposed reclassification of species within the genus *Neisseria*. Microbiology. 2012;158:1570-80

# Peer review: Reviewer response for version 1

**DOI:** 10.21956/wellcomeopenres.16741.r35816

**Published:** 2019-07-08

**Authors:** David S. Stephens, Yih-Ling Tzeng

**Affiliations:** 1 Division of Infectious Diseases, Emory University, Atlanta, GA, USA; 2 Emory Univeristy, Atlanta, GA, USA

Clemence, Harrison and Maiden report on the possible evolutionary origins of the meningococcal capsule locus. Based on whole genome sequencing (WGS) data acquired from a global collection of meningococcal and other *Neisseria spp* isolates, they propose the virulence critical capsule locus of *Neisseria meningitidis* was likely acquired *en bloc* by horizontal gene transfer (HGT) from *N. subflava. *This event has been followed by within-genes HGT recombination events including contributions from other *Nesseria* *spp*. and possibly other species that have given rise to the diversity of meningococcal capsule types. The authors had previously characterized putative capsule genes in non-pathogenic *Neisseria* *spp.* by WGS ^1^ and this is a follow up study based on new data. The report utilizes the genome data of the pubMLST database sited at the University of Oxford (now containing data from over 51,000 *Neisseria *family isolates) and the Meningitis Research Foundation meningococcus genome library among other genomic resources such as GenBank.

 Capsular polysaccharide is the major virulence factor resulting in the ability of *N. meningitidis* to cause invasive disease (e.g. meningitis and septicemia) in humans. Capsule is produced by a 24-30-kb genetic island, containing genes for capsule subunit and polymer biosynthesis, modification (i.e. acetylation), assembly, protection from degradation and transport. The meningococcal *cps* locus is divided into regions A, B, C, D, D’ and E, with B and C involved in capsule translocation and are the most conserved genes among different serogroups. The acquisition and evolution of the genetic island resulting in capsular polysaccharide assembly and transport is a key to understanding the emergence of meningococcal disease.

 The different capsule structures are the result of evolutionary divergent biosynthesis or polymerization genes found in Region A of the *cps* locus. Serologic typing and biochemical studies have classified the *N. meningitidis* capsular polysaccharides into a total of 13 serogroups (A, B, C, D, E, H, I, K, L, W, X, Y, and Z), with 12 (A, B, C, E, H, I/K, L, W, X, Y, and Z) confirmed genetically. Importantly, six capsular serogroups (A, B, C, W, X, and Y) currently cause almost all invasive meningococcal disease. These “invasive” capsules are composed of sialic acid homopolymers (serogroups B and C), alternating sialic acid with other glycans (serogroups Y and W), or repeating N-acetyl mannosamine-1-phosphate (A) or N-acetyl-d-glucosamine-1-phosphate (X).

 The origins of the meningococcal capsule locus have long been debated. Based on overall low GC content compared to the core meningococcal genome, the capsule cps locus has been designated a putative island of horizontally transferred DNA. Interestingly, the ~30-kb capsule genetic island has never been acquired (or has been lost) in the *Neisseria spp*. closely related to *N. meningitidis*, such as *N. lactamica, N. polysaccharea, N. cinerea* and the pathogen *N. gonorrhoeae*. A *cps* locus also is absent in the many commensal *N. meningitidis, *capsule null *(cnl) *meningococci, such as the members of clonal complexes cc198, cc53, cc192 *etc *.**that colonize the human nasopharynx *.*

 The capsule regions of other gram-negative bacteria, such as K1 *E. coli* and *H. influenzae* type b have important similarities in overall organization with the *cps *region of *N. meningitidis*[ref *-*2], suggesting a primordial capsule locus. However, there is a closer genetic relationship of regions of the capsule loci in the *Pasteurellacae* *(e.g. Pasteurella multocida*), *Actinobacillus* and *Kingella* families to the meningococcal *cps* locus. Recent work from Clemence et al. ^1^ found a *cps* locus in *N. subflava *and other more distantly related *Neisseria spp*. Further, in contrast to two or more recombination events, Brantley et al. ^3^ recently proposed that a single recombination event of the *cps* island into the *galE* allele of the *cnl* locus resulted in the formation of Region D’ containing the truncated *galE2* locus and the capture of the *cps* island *en bloc. * Retention of the duplicated Regions D and D’ enables the formation of a hairpin loop and inversion of the synthetic locus within the *cps* island during bacterial growth.

 In this report the authors use Neighbor-nets, BOOTSCAN and maximum likelihood phylogenies for genomic analysis. The authors found that the meningococcal cps regions B, C and D were highly mosaic. Sequences within regions B and C were phylogenetically nested within homologous sequences belonging to *N. subflava*, consistent with a HGT event in which *N. subflava *was the donor.

**Comments**:

 The report is generally well written, and the data provide additional clues to understanding the origins of the meningococcal capsule locus. The formation of meningococcal *cps* locus via HGT is a well-accepted concept. While the authors do not propose a paradigm-shifting conclusion, the data presented in the manuscript provided additional details as to the mosaic nature of the *cps* locus among different meningococcal isolates and the origins/donors of the HGT-mediated *cps* sequence compositions.

 Mobile elements e.g. IS *1016, *1S *1301* can shape the functionality of the meningococcal *cps* locus. For example, insertion and removal of IS *1301* in sialic acid producing meningococcal serogroups results in reversible off-on switching of capsule expression. IS *1301* insertion of *css*A also results in the loss of sialylation of LOS and recent studies have shown IS *1301* associated with large reversible inversions in the capsule locus ^4^. Was there any evidence that IS elements, Correia elements, and/or other mobile genetic elements are involved with the HGT events proposed between *N. subflava* and *N. meningitidis*?    

 Considering that the overall low GC content of the capsule *cps* locus compared to the core meningococcal genome has been used to designate this locus a putative island of horizontally transferred DNA, the authors should comment on the GC content difference between meningococcus and *N. subflava *in relation to the *en bloc* HGS conclusion.

 While the genomic relationships of the *rfb* and rfb’ genes to the *cps *locus is important, the functional role these genes play in *Neisseria spp.* including *N. meningitidis* and *N. gonorrhoeae* is not clear. While in other species they are involved in O-antigen (rhamnose synthesis) and LPS biosynthesis, that is not the case in *Neisseria spp*.

 Not well addressed in the report are data on the origins of the critical Region A biosynthesis genes that determine capsule structure and directly contribute to virulence. The possible origin of these genes should be better addressed. For example, the serogroup B polysaccharide capsule ([α2→8]-linked polysialic acid) is like the capsular polysaccharides of *Escherichia coli K1*, *Pasteurella haemolytica A2*, and *Moraxella nonliquefaciens*. What is the relationship of these genes to the meningococcal region A *cps* genes? Was the region A sequence similarly acquired in an en bloc fashion? Were the mosaic natures of region C and *galE *shown in BOOTSCANs of Figure 4 the result of subsequent HGS events providing the diversity of the region A?

 Please include the complete genus and species names. *Bibersteinia trehalosi* used as a comparison genome is a member of the genus of bacteria from the family of *Pasteurellaceae* and is a pathogen of sheep and can cause systemic infections in sheep. A. *A. actinomycetemcomitans* is now *Aggregatibacter actinomycetemcomitans.*

 Based on its origin, *N. weixii* seems an unlikely donor to the meningococcal gene pool. What other human *Neisseria* species are most closely related to *N. weixii*? Any matches in the human microbiome databases?

 The clarity of Figure 1. could benefit by including a diagram of the *cnl* locus. Also, showing in this figure the overall gene organizational schematics of the putative *cps* locus of *N. subflava* and *N. weixii* for a visual comparison with that of meningococcus would be useful.

We confirm that we have read this submission and believe that we have an appropriate level of expertise to confirm that it is of an acceptable scientific standard.

## References

[1] : Characterization of capsule genes in non-pathogenic Neisseria species. *Microb Genom*.4(9) : 10.1099/mgen.0.000208 10.1099/mgen.0.000208 30074474PMC6202450

[2] : Coating the surface: a model for expression of capsular polysialic acid in Escherichia coli K1. *Mol Microbiol*.1996;21(2) :221-31 885857810.1046/j.1365-2958.1996.6461357.x

[3] : Acquisition of the capsule locus by horizontal gene transfer in Neisseria meningitidis is often accompanied by the loss of UDP-GalNAc synthesis. *Scientific Reports*.2017;7(1) : 10.1038/srep44442 10.1038/srep44442 28290510PMC5349592

[4] : Emergence of a newNeisseria meningitidis clonal complex 11 lineage 11.2 clade as an effective urogenital pathogen. *Proceedings of the National Academy of Sciences*.2017;114(16) : 10.1073/pnas.1620971114 4237-4242 10.1073/pnas.1620971114 PMC540241628373547

# Response

**Published:** 2019-07-26

**Authors:** Marianne Clemence

**Affiliations:** University of Oxford, UK

We are grateful to the reviewers for their thorough analysis and interesting suggestions, and have now directly addressed them in version 2. A point-by-point response to their queries and suggestions follows:

 On insertion sequences, we did not specifically look for evidence that mobile genetic elements were involved in the HGT events between *N. subflava *and *N. meningitidis, *but none were identified during the annotation of the *N. subflava cps *in (1). Nevertheless, the reviewers raise an interesting point that may be worthy of investigation in future studies.

 With respect to GC content, *N. subflava* has ~50% GC content across the core genome, the same as *N. meningitidis,* therefore no inferences can be made about GC content in capsule export genes between the two species. As reported in (1), the putative region A of *N. subflava* is relatively low at 36-43%, similar to *N. meningitidis, *and is equivalently low in the majority of putative region A genes described in other non-pathogenic *Neisseria *species in the same study. Similarly, GC content has been reported to be low in the capsule synthesis region of other species (2–4). Therefore, whilst this is an interesting phenomenon with respect to the common ancestry of Gram-negative capsules, it cannot directly contribute to the present discussion. This has now been commented on in version 2 (discussion paragraph 4).

 In version two, we have adjusted introduction paragraph 2 to more accurately reflect current understanding of region D genes.

 The question of region A remains outstanding. Neither *N. subflava *nor *N. weixii* putative region A sequences, nor indeed any other putative non-pathogenic *Neisseria *putative region A, closely resemble any meningococcal region A. Despite the equivalence in biochemical structure between *E. coli *K1, *M. haemolytica *A2 and *M. nonliquefaciens *and meningococcal serogroup B (5), and the clear homology of the associated sequence regions, sequence identity has not been reported to exceed even 70% (6), yet the recombinant regions discussed in the paper appear to have diverged little from their donors. Therefore, we consider it unlikely, though not impossible, that these have directly donated sequences to the meningococcus. Our neighbour-net analyses and BOOTSCANs did not show any evidence that non- *Neisseria *sequences are found in *galE *or region C, so if they were a donor of region A, we contend that the breakpoints of such an event would lie within region A. The mosaic nature of region D does indicate HGT from an as yet unidentified species may exist, but this would be expected to be a member of the *Neisseria *(Figure 6B). In version two, we have added details (discussion paragraph 7). Our view is that we should be seeking novel *Neisseria *species, or species from closely related genera, to answer these questions.

 We have corrected the oversight about genus names in version two.

 We also acknowledge that *N. weixii *is an unlikely donor in paragraph two of the discussion, and in version two have corrected the last paragraph in the results that incorrectly implied that it was. As seen in Figure 2, this species is most closely related to *N. animalis*, which was isolated from a guinea pig (7), which is now discussed in paragraph two of the discussion in version 2. Based on cladistics, *N. mucosa, N. subflava*, *N. oralis, N. cinerea, ‘N. bergeri’, N. lactamica *and *N. polysaccharea *are equally closely related. We could not find any human-associated matches. Nevertheless, not all known human-associated species belong to a single clade. We will look with interest on any new human-associated *Neisseria* that might be discovered in the future.

 We have added a diagram of the *cnl *locus to paragraph one, in addition to schematics of the *cps *locus of *N. subflava *and *N. weixii *homologue, which are referred to in version 2 (results paragraph 1, discussion paragraph 5).

References

 1. Clemence MEA, Maiden MCJ, Harrison OB. Characterization of capsule genes in non-pathogenic *Neisseria species*. Microb Genomics. 2018;4(9).

 2. Clarke BR, Pearce R, Roberts IS. Genetic organization of the *Escherichia coli *K10 capsule gene cluster: Identification and characterization of two conserved regions in group III capsule gene clusters encoding polysaccharide transport functions. J Bacteriol. 1999;181(7):2279–85.

 3. Nielsen SM, de Gier C, Dimopoulou C, Gupta V, Hansen LH, Nørskov-Lauritsen N. The capsule biosynthesis locus of *Haemophilus influenzae* shows conspicuous similarity to the corresponding locus in *Haemophilus sputorum* and may have been recruited from this species by horizontal gene transfer. Microbiology. 2015;161(6):1182–8.

 4. Ito H. The genetic organization of the capsular polysaccharide biosynthesis region of *Actinobacillus pleuropneumoniae* serotype 14. J Vet Med Sci. 2015;77(5):583–6.

 5. Willis LM, Whitfield C. Structure, biosynthesis, and function of bacterial capsular polysaccharides synthesized by ABC transporter-dependent pathways. Carbohydr Res. 2013;378:35–44.

 6. Frosch M, Edwards U, Bousset K, Krausse B, Weisgerber C. Evidence for a common molecular origin of the capsule gene loci in Gram-negative bacteria expressing group II capsular polysaccharides. Mol Microbiol. 1991;5(5):1251–63.

 7. Berger U. *Neisseria animalis* nov. spec. J Hyg Infect Dis. 1960;147(2):158–61.
